# Optimization of the Biocatalysis for D-DIBOA Synthesis Using a Quick and Sensitive New Spectrophotometric Quantification Method

**DOI:** 10.3390/ijms21228523

**Published:** 2020-11-12

**Authors:** Gema Cabrera, Teresa Linares, Maria Elena de la Calle, Domingo Cantero, Antonio Valle, Jorge Bolivar

**Affiliations:** 1 Department of Chemical Engineering and Food Technology, Campus Universitario de Puerto Real, University of Cadiz, 11510 Puerto Real, Spain; gema.cabrera@uca.es (G.C.); elena.decalle@gm.uca.es (M.E.d.l.C.); domingo.cantero@uca.es (D.C.); 2 Institute of Viticulture and Agri-Food Research (IVAGRO)—International Campus of Excellence (ceiA3), University of Cadiz, 11510 Puerto Real, Spain; antonio.valle@uca.es; 3 Department of Biomedicine, Biotechnology and Public Health-Biochemistry and Molecular Biology, Campus Universitario de Puerto Real, University of Cadiz, 11510 Puerto Real, Spain; teresamaria.linares@uca.es; 4 Institute of Biomolecules (INBIO), University of Cadiz, 11510 Puerto Real, Spain

**Keywords:** whole-cell biocatalysis, D-DIBOA, spectrophotometric method, nitroreductase NfsB

## Abstract

D-DIBOA (4-hydroxy-(2H)-1,4-benzoxazin-3-(4H)-one) is an allelopathic-derived compound with interesting herbicidal, fungicidal, and insecticide properties whose production has been successfully achieved by biocatalysis using a genetically engineered *Escherichia coli* strain. However, improvement and scaling-up of this process are hampered by the current methodology for D-DIBOA quantification, which is based on high-performance liquid chromatographic (HPLC), a time-consuming technique that requires expensive equipment and the use of environmentally unsafe solvents. In this work, we established and validated a rapid, simple, and sensitive spectrophotometric method for the quantification of the D-DIBOA produced by whole-cell biocatalysis, with limits of detection and quantification of 0.0165 and 0.0501 µmol·mL^−1^ respectively. This analysis takes place in only a few seconds and can be carried out using 100 µL of the sample in a microtiter plate reader. We performed several whole-cell biocatalysis strategies to optimize the process by monitoring D-DIBOA production every hour to keep control of both precursor and D-DIBOA concentrations in the bioreactor. These experiments allowed increasing the D-DIBOA production from the previously reported 5.01 mM up to 7.17 mM (43% increase). This methodology will facilitate processes such as the optimization of the biocatalyst, the scaling up, and the downstream purification.

## 1. Introduction

**Figure 1:**
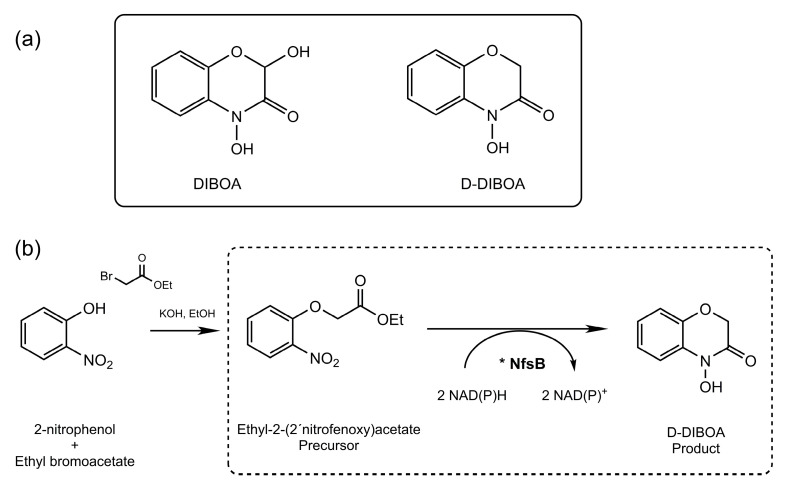
(**a**) DIBOA and D-DIBOA molecular structures and (**b**) D-DIBOA synthesis which involves two steps: The first step is a chemical reaction that using 2-nitrophenol starting material and ethyl bromoacetate to produce ethyl-2-(2′-nitrophenoxi)acetate (the precursor in the second step) by a nucleophilic substitution. The second step involves the reduction of a nitro group followed by a cyclization that is catalyzed by the nitroreductase (NfsB) enzyme (dashed box). This second reaction is catalyzed by NfsB nitroreductase which is NAD(P)H-dependent (*).

The use of allelochemicals from plants in agriculture is one of the most attractive strategies to avoid the negative impacts that weeds have on crops without harming the environment [1,2]. Benzohydroxamic acids are a group of these natural allelochemicals present in cereals such as wheat, rye, and maize [3]. These compounds are well known for their interesting biological properties as herbicides, fungicides, and insecticides [4]. Among them, DIBOA (2,4-dihydroxy-(2H)-1,4-benzoxazin-3-(4H)one) (Figure 1a) is considered an attractive model of natural herbicide [5] that shows high biodegradability in soils, and therefore, has a low environmental impact [6]. However, the main drawback of the use of DIBOA and other allelopathic compounds in agriculture is their reduced natural production [7]. An alternative is a chemical synthesis, a process that usually reaches relatively high yields [2]. In the case of DIBOA, the synthesis of the active analogous D-DIBOA (2-deoxy-DIBOA) is possible in a two-step sequence (Figure 1b) [2]. However, the scaling-up of this synthesis is hindered by the second step, which uses an expensive catalyst and is an exothermic reaction, with hydrogen release and a moderate molar yield (70%).

Nevertheless, the use of an engineered *Escherichia coli* strain overexpressing the autologous nitroreductase enzyme NfsB as a whole-cell biocatalyst can get over the drawbacks of the second step of this chemical synthesis [8]. Indeed, the engineered *E. coli* strain was capable of catalyzing the conversion of the precursor to D-DIBOA and exporting it out to the culture medium with a molar yield similar to that of the chemical synthesis. Furthermore, the optimization of the mutant background (*ΔlapAΔfliQ/*pBAD-NfsB strain), the use of an adjusted defined medium, and the addition of precursor in several loads made it possible to produce up to 5.01 mM D-DIBOA or 100% molar yield at lower concentration (4.4 mM) [9]. All these improvements carried out at laboratory scale encouraged us to tackle the optimization and the scaling-up of the biotechnological production of this biological herbicide. Nevertheless, one of the main bottlenecks to achieve these goals is the methodology for D-DIBOA quantification, which is currently based on high-performance liquid chromatography (HPLC). Although this technique is very accurate, it has several disadvantages when the measurement of a high number of samples or a rapid analysis is needed. HPLC requires expensive operation and instrumentation, and time-consuming sample processing. Besides, it also requires experience in the handling of the equipment [10].

Indeed, this is concerning drawback since the optimization and scaling-up of a bioprocess require intense experimental work going from laboratory scale through the pilot plant to the industrial implementation of the production. Therefore, it is essential to set up a fast and efficient analytical method to monitor D-DIBOA production in close to real-time, which would make it possible to carry out many assays at bioreactor scale and to achieve the automation of the process to obtain profitable yields and productivities.

Spectrophotometric methods are often used to quantify biological molecules because they allow quick and reliable ways to measure multiple biological samples. For this reason, classical methods for protein [11,12] or sugar [13] quantification are still routinely used, even though more accurate methodologies, such as mass spectrometry, have been tuned up in the last decades [14]. New spectrophotometric methods are still currently developed for fast detection of, for instance, environmentally toxic/hazardous substances such as pharmaceutical pollutants [15]. Particularly appealing is the application of this methodology for the quantification of compounds produced through biotransformation processes, since they allow the monitoring of the procedure, which helps to optimize the production [16].

**Figure 2:**
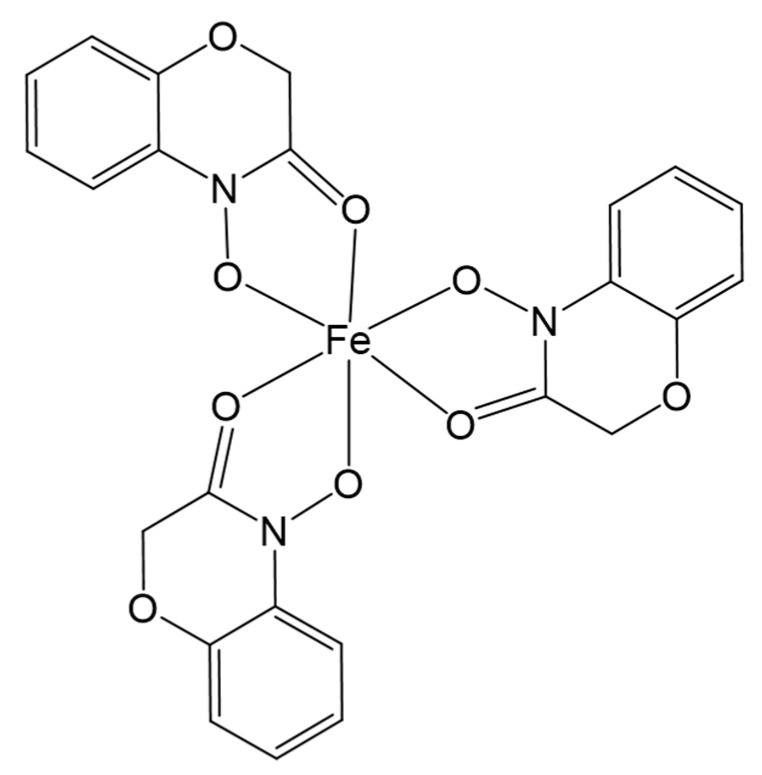
Molecular structure of the complex Fe (III)-(D-DIBOA)_3_.

Ferric chloride (FeCl_3_) has been previously used for organic compound determination because hydroxamic acids typically coordinate iron (III) with high affinity in acidic-alcoholic FeCl_3_ solutions and generate blue colored complexes [17], which allows their detection [18,19,20]. Nonetheless, in these studies, the acidic-alcoholic FeCl_3_ solution was applied to plant or soil natural extracts containing a mix of several hydroxamic acids, all of which contributed to the development of colored complexes with Fe (III). For this reason, this technique was replaced by more selective and sensitive methodologies such as gas chromatography (GC) coupled to mass spectrometry (MS) and liquid chromatography (LC) coupled to ultraviolet (UV) detection, or the even more sensitive LC coupled to MS and tandem MS. These techniques played essential roles in the detection, separation, and quantification of natural benzohydroxamic acids isolated from plant tissues or their degradation products in the soil [21]. Nevertheless, in the case of the whole-cell biocatalysis the sample contains a single benzohydroxamic acid (D-DIBOA). We hypothesized that a complexation reaction of D-DIBOA with Fe (III) in an acidic medium would generate a blue-colored Fe (III) hydroxamate complex that could be useful for the quantification of our target product (Figure 2). It is worth noting that the precursor for D-DIBOA synthesis does not contain the hydroxylamine moiety and would presumably not react with Fe (III) (Figure 1).

The methods previously described for the detection of natural benzohydroxamic acids (DIBOA, DIMBOA) could not be applied directly because they used ethyl acetate extracts from plants or soil and an acidic-alcoholic FeCl_3_ solution to generate the colored complexes [18]. However, in the whole-cell biocatalysis, D-DIBOA, which had not been previously tested for this reaction, is dissolved in an aqueous solution (the bacterial culture medium). Besides, this alternative method should be as reliable as HPLC for the quantification of this compound, and also has to be sensitive enough to quantify D-DIBOA, at least, in the range of millimolar concentration.

To explore the feasibility of this approach, the maximum wavelength absorbances, concentrations, and compositions of reagents were established in order to carry out the reaction in an efficient and economical manner. The possibility of using low volumes was also explored with the aim of quantifying multiple samples in a microtiter reader plate. Finally, the method was validated by calculating different parameters such as linearity, accuracy, precision, and sensitivity, in addition to the limit of detection (LOD) and the limit of quantification (LOQ). Validation was carried out in accordance with the International Conference on Harmonization (ICH) rules Q2 (R1) [22].

The applicability and utility of the methodology was also tested on real biotransformation assays. To this end, we performed several biocatalytic strategies by monitoring D-DIBOA using the *ΔlapAΔfliQ*/pBAD-NfsB strain [9].

## 2. Results

### 2.1. Development of a Spectrophotometric Method for D-DIBOA Quantification

#### 2.1.1. Assessment of Maximum Wavelength (λ_max_) Range for Absorbance of the Fe (III)-(D-DIBOA)_3_ Complex

**Figure 3:**
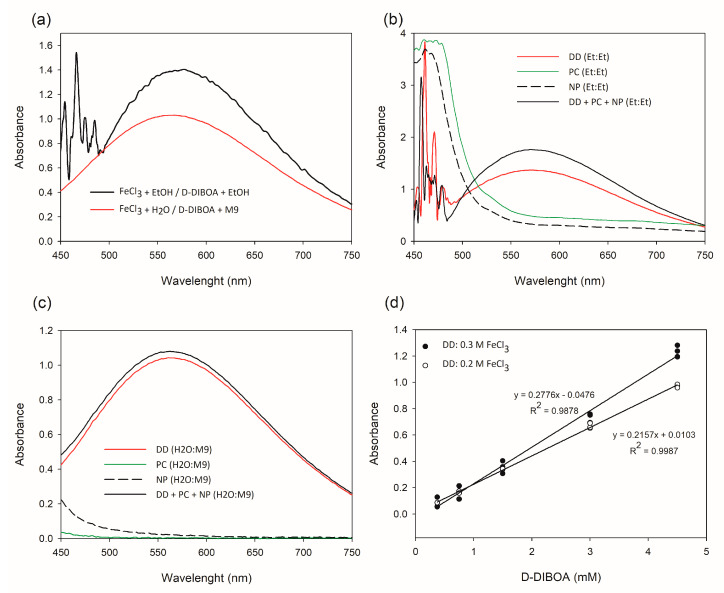
Spectral absorbance determination of Fe (III)-(D-DIBOA)_3_ complex, specificity, and optimization of calibration curves. (**a**) Spectral absorbance of Fe(III)-(D-DIBOA)_3_ complex in ethanol and M9 medium; (**b**) spectral absorbance curves of: D-DIBOA (DD), precursor (PC), 2-nitrophenol (NP) dissolved in ethanol (Et), in the presence of FeCl_3_ also dissolved in Et; (**c**) spectral absorbance curves of: D-DIBOA, precursor, 2-nitrophenol dissolved in M9, in the presence of FeCl_3_ dissolved in water; (**d**) calibration curves of D-DIBOA (mM) by adding FeCl_3_ 0.2 and 0.3 M with pH < 1 and measured spectrophotometrically at a wavelength of 570 nm in a microplate reader.

To assess the λ_max_ absorbance of the complex Fe (III)-(D-DIBOA)_3_, 2 mM D-DIBOA was used to scan the visible range (OD_450–750_) with different solvents for D-DIBOA and FeCl_3_. We first tested a method previously described to detect hydroxamic acids [18], in which the samples were dissolved in ethanol and the FeCl_3_ (0.37 M) solution was prepared in acidic ethanol. Nevertheless, this method cannot be directly applied to D-DIBOA biocatalysis because the target molecule is dissolved in an aqueous solution. For this reason, we also assayed the same FeCl_3_ concentration in acidic water (pH < 1) for testing D-DIBOA dissolved in M9 culture medium (the same matrix in which D-DIBOA is produced). The combination of D-DIBOA in M9 and FeCl3 in ethanol was discarded to avoid salt precipitation. The range of λ_max_ measured in both conditions was 550–600 nm, although the ethanol dissolved solution also gave irregular results at wavelengths below 500 nm (Figure 3a).

#### 2.1.2. Specificity

Specificity is a key issue for quantification. In our case, this parameter was evaluated by comparing the scanning spectra of a D-DIBOA solution with and without the two organic compounds from the biocatalysis that may also be present in the culture media, that is, 2-nitrophenol (as the unreacted starting material of the first step of the chemical synthesis) and the precursor of D-DIBOA synthesis. As can be observed in Figure 3b, both 2-nitrophenol and the precursor showed absorbance in the entire visible spectrum when the acidic-ethanol FeCl_3_ solution was applied, interfering with the recovery of D-DIBOA. These interferences were negligible when the acidic water-FeCl_3_ solution was used, mainly from O.D. 560 on (Figure 3c). This reactant has the additional advantage of avoiding the use of ethanol, an expensive organic solvent. An acidic water-FeCl_3_ solution and a wavelength range of 560–600 nm were therefore selected for further implementation of the method.

#### 2.1.3. Optimization of the Calibration Curve

A spectrophotometric quantification method requires reliable linearity in the calibration curve in the range of concentrations to be measured. To investigate whether the absorbance of Fe(III)-(D-DIBOA)_3_ complex could be used toward this aim, we tested several FeCl_3_ solutions (0.37, 0.3, 0.2, and 0.1 M; pH < 1) to optimize the conditions for the maximum absorbance and linearity. We first scanned the visible spectrum with the four FeCl_3_ concentrations and a D-DIBOA 2 mM solution, and we found out that applying 0.1 M FeCl_3_ did not give differences in OD_450–750_, probably because the reactant concentration was too low. On the other hand, 0.37 and 0.3 M showed similar intensities Figure S1). We therefore selected 0.3 and 0.2 M FeCl_3_ to establish a calibration curve, using five standard solutions containing D-DIBOA from 0.375 to 4.500 mM dissolved in M9 medium and analyzed the linearity of the standard curves by calculating the correlation coefficient (R^2^) between intensity and concentrations (Figure 3d). Although the applications of both 0.3 and 0.2 M FeCl_3_ showed absorbance readings in the range of the D-DIBOA solutions tested, the use of 0.2 M FeCl_3_ gave the best correlation (R^2^ = 0.9987). The slope (0.2157) indicates a high degree of sensitivity, sensitivity being the capacity of the method to distinguish, with a determined degree of reliability, two proximal concentrations (Figure 3d). Additionally, the 0.2 M FeCl_3_ solution was more stable than the 0.3 M one, since we observed some precipitated FeCl_3_ 24 h after the preparation of the latter solution (data not shown). In these experiments, the volume of the reaction was scaled down to 0.2 mL using a microtiter reader plate at OD_570_. This latest optimization reduced the amount of reagent and sample required in the quantification, which is relevant for whole-cell biocatalysis assays at bench scale (100 mL). Additionally, it allows the quantification of hundreds of samples in a short time.

In summary, we have been able to establish an economical, low time consuming, and sensitive method for the quantification of biotechnologically produced D-DIBOA by using 100 µL FeCl_3_ dissolved in acidic water (0.2 M, pH < 1) and 100 µL of a D-DIBOA sample dissolved in M9 medium, and directly measured at the wavelength of 570 nm in a microtiter reader plate.

#### 2.1.4. Method Validation

The validation of the spectrophotometric analytical method was carried out on the basis of the following parameters:

Sensitivity: The limit of detection (LOD) and limit of quantification (LOQ) values were 0.0165 and 0.0501 mM, respectively; both being values below the lowest concentration of the linear range (Table 1). The results indicated that the method is sensitive even at low concentrations (in the range of 50 µM D-DIBOA concentrations).

Accuracy: The accuracy of the method was determined by recovery test of known quantities of D-DIBOA. The values obtained were in the range of 98.24–102.22%, which are into the acceptable range since the values showed standard deviations less than 2.5% (Table 1 and Table 2).

Precision: Inter-analyst analysis showed the higher relative standard deviations (RSD), with values from 2.4372 to 4.2308% for the highest and the intermediate concentrations. The rest of the values ranged from 0.9505 up to 2.2833% for method data in the cases of inter-day and intra-day. In addition, since the Horrat parameter is less than 2 in all the cases, it can be assumed that the method has satisfactory reproducibility values (Table 3) [23].

Reagent stability: The FeCl_3_ solution stability was evaluated for 4 days, by comparing D-DIBOA quantification prepared on day 1 with a fresh prepared solution. No statistically significant differences were found between the sets of experiments (data not shown).

#### 2.1.5. Method Reliability

**Figure 4:**
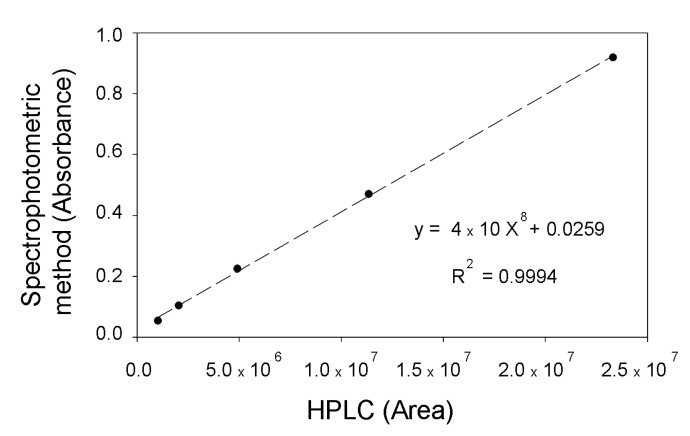
Correlation of OD_570_ using the D-DIBOA spectrophotometric method developed in this work versus the HPLC analysis (peak areas) in the method previously established (standards 0.25–4 mM).

In order to further validate the spectrophotometric method, the correlation between this analysis and the HPLC one was studied by comparing the recovery percentage in both cases using samples from different standards, conditions, and experiments. The adjustment between both methodologies was carried out through regression coefficient calculation (R^2^), obtaining a value of 0.9994 (Figure 4). These results show that, in the assayed conditions, the spectrophotometric method is as reliable as the HPLC one and it can therefore be used to measure D-DIBOA concentration at close to real-time range.

### 2.2. Optimization of the Whole-Cell Biocatalysis for D-DIBOA Using the Spectrophotometric Quantification Method

The applicability of the spectrophotometric method was analyzed by monitoring D-DIBOA concentration in 100 mL biocatalysis assays carried out at bench scale started with the addition of an initial precursor load to reach 2.2 mM in the culture medium, as described in the method section. The aim of these experiments was to monitor D-DIBOA concentration at real time, by applying the method proposed in this work every hour from 2 to 12 h and at 24 h as final time point. This also allowed us to stoichiometrically calculate the presence of unreacted precursor. This is a key element to optimize D-DIBOA production, since precursor concentrations above 2.2 mM inhibited the bacterial biocatalyst [9].

**Figure 5:**
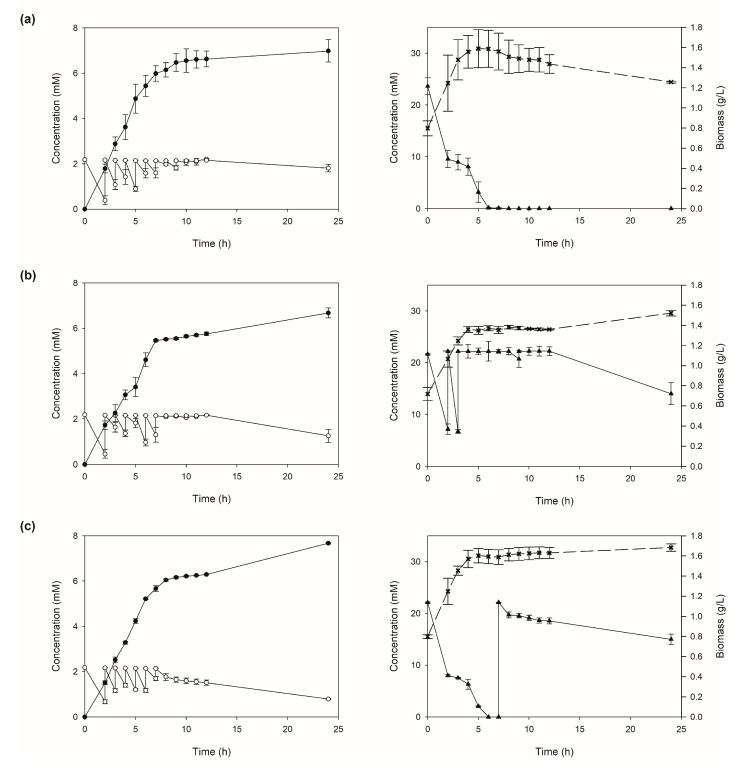
Plots of biotransformation experiments for the optimization of D-DIBOA production. The left panels show D-DIBOA concentrations (mM) quantified by the spectrophotometric method (black circles) and precursor concentrations calculated stoichiometrically (white circles). In the right panels are shown plots of biomass (cross) and glucose concentrations (triangles). (**a**) Biotransformation assays keeping the precursor concentration at 2.2 mM by adding a fresh load every hour (from 2 to 10 h) with no glucose addition; (**b**) biotransformations with the same precursor loading strategy but keeping the glucose concentration at 22.2 mM every hour (from 2 to 10 h); (**c**) biotransformation assays keeping the precursor concentration at 2.2 mM by adding a fresh load every hour from 2 to 7 h and a single glucose pulse at 7 h to reach 22.2 mM.

This series of biotransformation assays was designed as a proof of concept for the application to the scaling up of D-DIBOA production by monitoring not only D-DIBOA and precursor concentrations (left panels in Figure 5), but also other fundamental parameters, such as bacterial growth and glucose consumption (right panels in Figure 5) and NH_4_^+^ consumption. Thus, precursor concentrations were estimated at each time point and then a precursor load was added to reach 2.2 mM (inhibitory precursor concentration) at time points from 2 to 12 h. This strategy allowed us to improve the D-DIBOA production from the previously reported 5.01 mM up to 6.80 mM (Figure 5a, left panel), although around 20% of the precursor added was not biotransformed. It is worth noting that these experiments also showed that D-DIBOA production is associated with cell growth (Figure 5a, right panel) since the maximum biotransformation rate was found at log phase (from 2 to 7 h). On the other hand, from 7 to 24 h, cell growth not only remained in stationary phase, but also even decreased and D-DIBOA production nearly stopped.

Glucose concentration in the culture medium was also associated to the biocatalysis because it was almost totally consumed at 6 h (Figure 5a, right panel). We reasoned that this could be the cause of cell growth inhibition. Therefore, in another series of experiments (Figure 5b), glucose loads were applied to maintain the initial glucose concentration (22.2 mM). However, cell growth did not improve up to 6 h despite the fact that glucose was available for the cells; on the contrary, cell growth and D-DIBOA production slightly decreased with respect to the previous experiments. Nevertheless, from 12 to 24 h cells kept growing at a low rate (Figure 5b, right panel), and consistently, D-DIBOA concentration increased (Figure 5b, left panel), although the final production was virtually the same as in the assays with no glucose reloading (6.70 mM). We therefore focused our efforts on improving molar yields (Figure 5c). In order to do so, the last precursor load was added at the end of the log phase (7 h after the initial load). We also controlled glucose concentration to improve cell growth by not adding glucose during the log phase but adding a single load at hour 7 to maintain cell growth for the rest of the assay (Figure 5c, right panel). This strategy increased D-DIBOA production up to 7.17 mM (Figure 5c, left panel), transforming 89.90% of the precursor, and therefore increasing the molar yield. In addition, the bacteria growth slightly increased under these conditions (Figure 5c, right panel).

### 2.3. Tolerance of the E. coli ΔlapAΔfliQ/pBAD-NfsB Strain to D-DIBOA

**Figure 6:**
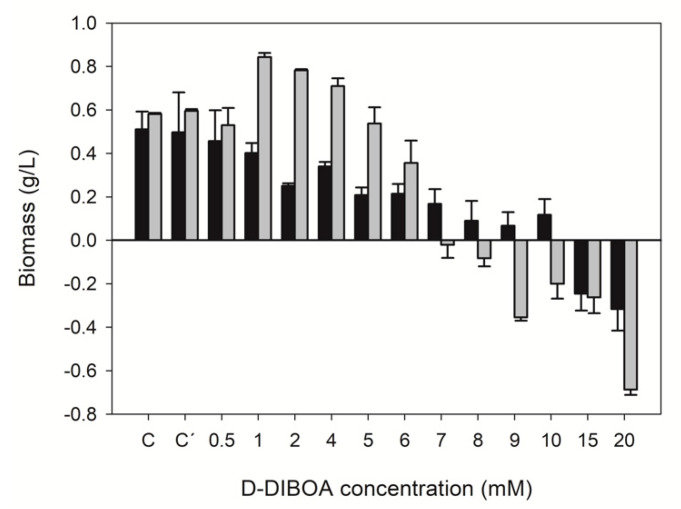
Tolerance test on BW25113/pBAD-NfsB (black bars) and *ΔlapAΔfliQ*/pBAD-NfsB (grey bars) *Escherichia coli* strains for increasing D-DIBOA concentrations. Bar charts show relativized values of bacterial biomass grown for 12 h with respect the biomass at the moment in which D-DIBOA was added, in an OD_600nm_ = 0.6. Bacterial growth was measured in absence (positive control, C; and positive control with MeOH, C´) or in the presence of different concentrations of D-DIBOA. The error bars represent the standard deviations of three independent replicates.

As indicated in the previous section, D-DIBOA bioproduction seems to reach a maximum theoretical concentration at around 7 mM on this strain under the conditions tested in this work. In order to elucidate whether the cause of this threshold is a toxic effect of D-DIBOA on the bacterial strain, we studied the tolerance to D-DIBOA on the optimized Δ*lapAΔfliQ*/pBAD-NfsB and the parental (BW25113/pBAD-NfsB) strains by analyzing biomass growth on M9 medium containing increasing D-DIBOA concentrations. The results of this assay show that the optimized strain was more tolerant to D-DIBOA than the parental one in D-DIBOA concentration range of 1–6 mM. However, the parental strain was able to grow under concentrations between 7 to 10 mM of D-DIBOA, while Δ*lapAΔfliQ*/pBAD-NfsB strain did not grow at 7 mM and even provoked cell lysis at higher concentrations (Figure 6). We concluded therefore that 7 mM was the limiting toxic D-DIBOA concentration for this strain.

## 3. Discussion

Whole-cell biocatalysts provide unique advantages and have been widely used for the efficient biosynthesis of value-added fine and bulk chemicals, and pharmaceutically active ingredients [24]. We previously used this approach to produce D-DIBOA, a synthetic analogous to the natural allelopathic herbicide DIBOA (Figure 1a). This benzohydroxamic acid present in common crops, such as wheat, rye, and maize, is a promising natural herbicide model, showing high biodegradability in soils, and therefore, a low environmental impact [2]. However, the main drawbacks for the use of DIBOA and other allelopathic compounds in agriculture are their very low natural production and the complexity of their biosynthetic pathways, which involve multiple enzymes, making them difficult to be translated to microorganisms for their biotechnological production. The chemical synthesis of the analogous D-DIBOA is a simpler process that involves only two steps and uses inexpensive starting materials, although the second step, a difficult and expensive process, is a bottleneck for the industrial production.

This drawback turns, therefore, a green process—the production of an environmentally friendly herbicide—into an unsustainable and economically unfeasible process. A way to overcome this problem is biocatalysis, which is based on the specificity and high catalytic capability of enzymes. Biocatalytic processes also meet the requirements of green chemistry because they are carried out in aqueous solutions, mild temperatures, and low pressures, and consequently lead to less waste than chemical syntheses. A biocatalyst can be a cell extract or a purified enzyme used in vitro, either in solution or immobilized [25]. However, living microorganisms can also be used as whole-cell biocatalysts, taking advantage of the capacity of a cell to transform an external precursor into the target chemical compound.

We previously reported that *E. coli* could be effectively used as a whole-cell biocatalyst to replace the second reaction of D-DIBOA chemical synthesis by over-expressing the autologous nitroreductase NfsB [9]. Furthermore, this versatile system also allows the production of D-DIBOA chlorinated derivatives [26].

Nevertheless, these bacterial biocatalysts can be potentially further improved by genetic manipulation of the genetic background. For instance, screening of mutant collections has shown to be a feasible strategy [9]. Other improvements could be based on adaptive evolution approaches in order to find *E. coli* strains more tolerant to limiting factors such as high precursor and D-DIBOA concentrations. On the other hand, the process also needs to be scaled up from an Erlenmeyer flask to higher volume bioreactors in which the operation mode must be adjusted and automated if possible. Additionally, last but not least, D-DIBOA must be purified from the culture medium. These downstream procedures are often some of the most expensive and difficult goals to achieve for the industrial application. All these processes require multiple experiments that require a reliable but also a fast and inexpensive D-DIBOA quantification. The current methodology based on HPLC techniques does not meet these requirements. For instance, the screening of mutants carried out in our laboratory was limited to a relatively low number of strains, mainly due to the expensive and time-consuming D-DIBOA quantification.

For this reason, before tackling further improvements of the bioprocess, it was compulsory to establish a faster, easier, and cheaper quantification method. We successfully achieved this goal by implementing and validating a spectrophotometric method based in the generation of blue colored Fe(III)-(D-DIBOA)_3_ complexes (Figure 2). This method allows an accurate quantification in the range of millimolar concentration in the M9 culture medium used for D-DIBOA bioproduction. An extensive validation of the method demonstrates that it is as reliable as HPLC in our experimental conditions, but in contrast to the chromatographic techniques, it can be carried out in a real time manner using microtiter plates and consuming only 100 μL of sample in the quantification. The equipment and chemicals required are simple and easy to use, allowing the processing of hundreds of quantification tests per hour.

As a proof of concept, this method was applied to real biotransformation experiments at laboratory scale. The monitoring of precursor and D-DIBOA using the methodology proposed in this work at several time points allowed us to increase D-DIBOA production up to 7.17 mM with 89.9% of molar yield, and disclosed relevant information about the biocatalysis that could not otherwise be found (Figure 5). For instance, we found that D-DIBOA production is closely related to cell growth and the limiting D-DIBOA concentration of 7 mM for the growth in the Δ*lapA*Δ*fliQ*/pBAD-NfsB strain (Figure 6).

## 4. Materials and Methods

### 4.1. Bacterial Strains

The *E. coli ΔlapAΔfliQ*/pBAD-NfsB strain was used as whole-cell-biocatalyst and BW25113/pBAD-NfsB as wild type strain This strain harbors deletions of the genes encoding for the lipopolysaccharide assembly protein A (*lapA*) and the flagellar biosynthesis protein (*fliQ*); and the construct pBAD-NfsB [8] that allows the controlled overexpression of the nitroreductase NfsB upon addition of l-arabinose to the culture medium. This engineered strain was constructed for optimization of D-DIBOA yield and concentration as described previously [9] and it was registered in the Spanish Type Culture Collection (CECT 9760).

### 4.2. Culture Media and Chemicals

The strains used in this work were initially grown in Luria–Bertani (LB) medium and LB agar plates. For the biotransformation tests, M9 minimal medium was used, containing (g/L): 0.24 MgSO_4_, 0.01 CaCl_2_, 11.178 Na_2_HPO_4_, 3.00 KH_2_PO_4_, 0.50 NaCl, 1.00 NH_4_Cl, and 4.00 glucose. Chemicals for the culture media were purchased from Panreac (Barcelona, Spain). The medium was supplemented with 50 µg/mL kanamycin and 100 µg/mL ampicillin when appropriate. NfsB overexpression in the pBAD/His-A vector was induced by adding 0.02% (*w/v*) l-arabinose. Antibiotics and l-arabinose were purchased from Sigma-Aldrich. D-DIBOA and its synthetic precursor—the ethyl-2-(2′-nitrophenoxy)acetate—were obtained by chemical synthesis kindly provided by Allelopathy Group Organic Chemistry Department, University of Cadiz [2]. Starting material (2-nitrophenol) and other chemicals such as ethyl bromoacetate (EtBr), methanol (MeOH), ethanol (EtOH), ferric chloride (FeCl_3_·6H_2_O), and hydrogen chloride (HCl), were purchased from Panreac Quimica (Barcelona, Spain). Biological samples of D-DIBOA were obtained by biocatalysis in our laboratory.

### 4.3. Biotransformation Assays

The bacterial strains were streaked from a −80 °C glycerol stock on LB agar plates and incubated overnight at 37 °C. A single colony was inoculated in 5 mL LB medium and cultivated at 37 °C and 200 rpm in an orbital shaker. After 8 h, the cells were centrifuged at 3000× *g* for 10 min and the pellet was resuspended in 100 mL M9 medium containing 0.02% (*w/v*) l-arabinose, and then it was incubated overnight in the same conditions; 10 mL of this pre-culture was then centrifuged at 3000× *g* for 10 min and resuspended in 100 mL of fresh M9 medium supplemented with 0.02% (*w/v*) l-arabinose and grown in a 250 mL Erlenmeyer flask at 37 °C. The biotransformation assay was initiated by adding 1 mL of precursor stock solution (50 mg/mL in 100% MeOH) when OD_600_ arose 0.6; then the precursor concentration was therefore 0.5 mg/mL (2.22 mM), which was denoted as time point 0 h. In order to enhance the D-DIBOA titer, successive loads of precursor were done, keeping the concentration at 2.2 mM over the experiment. Another parameter studied was the carbon source; therefore, successive loads of glucose were also maintained constant at 22.2 mM. For both assays, the rapid quantification of D-DIBOA method studied in this work was applied. All of the experiments were carried out in triplicate.

### 4.4. Chemicals for Benzohydroxamic Acid Determination and Quantification

Stock solutions (200 mM) of D-DIBOA, 2-nitrophenol, and precursor were prepared in 100% methanol. FeCl_3_ solutions were prepared by dissolving FeCl_3_ in ethanol or water and the pH was adjusted by adding HCl (1.5 M). In the case of D-DIBOA obtained by biotransformation, an appropriated volume of the bacterial cell culture was centrifuged at 10,000× *g* for 10 min and the supernatant was used for quantification.

### 4.5. Analytical Instruments and Techniques, Calculation of Parameters, and Statistical Analysis

A UV–visible spectrophotometer (U-2001 spectrophotometer HITACHI Instruments Inc. Tokyo, Japan) was used to scan the absorbance spectra in the visible range of wavelengths (400–750 nm) in order to select the maximum wavelength of Fe(III)-(D-DIBOA)_3_ complex absorption. Multiskan FC^®^ microtiter reader plate with incubator (Thermo Scientific, Thermo Fisher Scientific, Madrid, Spain) was used for D-DIBOA quantification in the experiments related to the method validation and in the whole-cell biocatalysis assays. Precursor and D-DIBOA were additionally measured in some experiments by reverse-phase high performance liquid chromatography (HPLC) (Merck HITACHI HPLC system, Tokyo, Japan) equipped with Phenomenex Gemini C18 4.6 × 250 mm column (Torrace, CA, USA), using the method previously described [8]. Samples were filtered through 0.22 µm nylon filters (VWR International, Barcelona, Spain) before HPLC analysis.

Cell growth was estimated by measuring OD_600_ by the ratio, 1 OD_600_ = 0.33 g of cell dry weight (CDW)/L, according to standard procedure [27]. Samples were centrifuged at 11,000× *g* for 5 min in order to remove biomass before analysis of the rest of the parameters. Glucose concentration was determined by using the glucose oxidase and peroxidase enzymatic Kit (BioSystems, Barcelona, Spain) and measured in a microtiter reader plate at 425 nm wavelength.

Biotransformation yield (BY) was calculated from the concentration of D-DIBOA at the biotransformation time assayed, and we the initial precursor concentration:

(1) $$\mathrm{BY}\;=\frac{\mathrm{mol}\;\mathrm{DDIBOA}}{{\mathrm{mol}\;\mathrm{precursor}}_{i}}\;\times 100\;\left(\%\right)$$

Average and standard deviation were calculated using at least 3 replicates.

For the biotransformation assays, precursor concentration in the culture medium (mM) was stoichiometrically calculated for each time point (t) as follows:

(2) $$\left[\mathrm{Precursor}\right]t\;=\;\frac{\mathrm{mmol}\;\mathrm{precursor}\;\mathrm{added}\;\left(t\right)-\mathrm{mmol}\;\mathrm{DDIBOA}\;\mathrm{measured}\;\left(t\right)}{1L\;\mathrm{biocatalysis}\;\left(t\right)}$$

### 4.6. Method Validation

The method for D-DIBOA quantification was validated by taking into consideration the following parameters: specificity, linearity, limit of detection (LOD), limit of quantification (LOQ), accuracy, precision, and robustness. Validation of these parameters was applied according to the International Conference on Harmonization (ICH) guidelines Q2 (R1) [22] and was calculated as follows:

Specificity: It was evaluated by comparing the visible spectra of standard solutions of D-DIBOA against other compounds that can be present in the biotransformation culture. The analysis scanning was performed from 450 to 750 nm, checking for changes in absorbance.

Linearity was analyzed by quantifying three different series of five D-DIBOA standard solutions (0.375, 0.75, 1.5, 3, 4.5 mM), analyzed in triplicate, and plotted in nine derived analytical curves. The linearity was verified by linear regression analysis using the least squares method (R^2^).

Limit of detection (LOD) and limit of quantification (LOQ) were determined in order to establish the sensitivity of the developed method. Both parameters were calculated by LOD = 3.3(S.D/S) and LOQ = 10(S.D/S) where SD is the standard deviation of the response of the blank and S is the slope of the analytical curve. Blanks were prepared in M9 medium.

Accuracy was examined by the recovery of a known amount of D-DIBOA added to M9 medium. For that, three different levels of analyte concentration were used: lower concentration (0.75 mM), intermediate concentration (1.5 mM), and higher concentration (3.0 mM). Samples were prepared in triplicate and analyzed using the proposed method. The relative standard deviation (RSD) and D-DIBOA recovery percentage were used to evaluate the accuracy of the method applied.

(3) $$\mathrm{Recovery}\;\left(\%\right)=\frac{\mathrm{theoretical}\;\mathrm{analyte}\;\mathrm{concentration}}{\mathrm{found}\;\mathrm{analyte}\;\mathrm{concentration}}$$

Precision was verified by evaluating replicability and intermediate precision (inter-day, intra-day, and inter-analyst), while analyzing three different levels of D-DIBOA (0.75, 1.5, and 3.0 mM) in triplicate. Analyses were carried out for two different analysts at two different times during one day for intra-day precision evaluation. The same procedure was followed in a second day to study inter-day variations. Firstly, the RSD was calculated; then the values obtained were analyzed by the use of the Horrat parameter, to give a measure of the acceptability of the method precision, as described below [23]:

(4) $$\mathrm{Horrat}=\;\frac{{\mathrm{RSD}}_{R}}{{\mathrm{RSD}}_{H}},\;\mathrm{where}$$

(5) $${\mathrm{RSD}}_{R}\;\mathrm{is}\;\mathrm{the}\;\mathrm{real}\;\mathrm{relative}\;\mathrm{standard}\;\mathrm{deviation}\;\mathrm{CV}\;\left(\%\right)=\frac{S.D}{\mathrm{Average}}\times 100;$$

(6) $${\mathrm{RSD}}_{H}\;\mathrm{is}\;\mathrm{the}\;\mathrm{predicted}\;\mathrm{value}\;\mathrm{obtained}\;\mathrm{from}\;\mathrm{the}\;\mathrm{Horwitz}\;\mathrm{equation}:\;2^{\left(1-0.5\mathrm{logC}\right)}\left(\%\right),\;\;\mathrm{where}\;C\;\mathrm{is}\;\mathrm{the}\;\mathrm{concentration}\;\mathrm{of}\;\mathrm{the}\;\mathrm{analyte}.$$

## 5. Conclusions

In this work we have developed a spectrophotometric method for the quantification of the benzohydroxamic acid D-DIBOA when this compound is dissolved in the defined culture medium. This method shows high accuracy, precision, and sensitivity. It also shows a high correlation coefficient (0.9994) with the HPLC quantification method and can be carried out using a low volume of sample on a microtiter plate reader. This method allowed us to increase 43% D-DIBOA production and will help to design the operation mode for the scaling up of the D-DIBOA biotechnological production, and will also facilitate the research on its purification from the culture medium. This approach also could be applied to the production of others benzohydroxamic acids, such as the chlorinated D-DIBOA derivatives.

## Figures and Tables

**Table 1:** Analytical parameters for the complex Fe(III)-(D-DIBOA)_3_.

Parameter	Range
λ_max_	570 nm
Beer´s law range	0.75–3 mM
Slope	0.2181
Intercept	0.0104
Correlation coefficient	0.9997
Accuracy	98.24–102.22%
Precision (%RSD)	0.95–2.07%
LOD	0.0165 mM
LOQ	0.0501 mM

**Table 2:** Recovery percentage of D-DIBOA in M9 medium to evaluate the accuracy of spectrophotometric method.

Added D-DIBOA (mM)	Detected D-DIBOA Mean ± SD ^a^ (mM)	Mean Recovery (%) ± SD ^a^ (mM)
0.75	0.7368 ± 0.0090	98.24 ± 1.20
1.5	1.5451 ± 0.0149	101.67 ± 1.00
3	3.0667 ± 0.0645	102.22 ± 2.15

^a^ Indicates the mean and standard deviation (SD) of nine replicates (*n* = 9).

**Table 3:** Precision of proposed analytical method. Concentration found and recovery percentage of D-DIBOA in M9 medium to evaluate the precision for inter-day, intra-day and inter-analyst.

Precision	Added (µmol·mL^−1^)	Found (µmol·mL^−1^) ± SD ^a^	RSD_R_ (%)	Horrat Parameter
Method				
	0.75	0.7368 ± 0.0090	1.1476	0.2345
	1.5	1.5251 ± 0.0149	0.9505	0.2117
	3	3.0667 ± 0.0645	2.0697	0.4998
Inter-day				
Same analyst, day 1 and day 2	0.75	0.7347 ± 0.0141	2.2833	0.4666
	1.5	1.5270 ± 0.0204	1.1055	0.2463
	3	3.0412 ± 0.0532	1.8961	0.4579
Intra-day				
Same analyst, day 1, different test	0.75	0.7373 ± 0.0111	2.1773	0.4449
	1.5	1.5283 ± 0.0224	1.1790	0.2626
	3	3.0698 ± 0.0456	1.7387	0.4198
Inter-analyst				
Analyst 1 and 2, day 1	0.75	0.7646 ± 0.0180	3.2860	0.6715
	1.5	1.5973 ± 0.0818	4.2308	0.9424
	3	3.0908 ± 0.0886	2.4372	0.5885

^a^ Indicates the mean and standard deviation (SD) of nine determinations (*n* = 9).

## Supplementary Materials

Supplementary materials can be found at https://www.mdpi.com/1422-0067/21/22/8523/s1.

## Abbreviations

CV	coefficient of variation
DIBOA	2,4-dihydroxy-(2H)-1,4-benzoxazin-3(4H)-one
D-DIBOA	4-hydroxy-(2H)-1,4-benzoxazin-3(4H)-one
HPLC	high performance liquid chromatography
LOD	limit of detection
LOQ	Limit of quantification
NfsB	NAD(P)H-dependent nitroreductase
RSD	relative standard deviation
