# Supplementary material for: Optimization of the Biocatalysis for D-DIBOA Synthesis Using a Quick and Sensitive New Spectrophotometric Quantification Method
Source: Int J Mol Sci. 2020 Nov 12;21(22):8523. doi: 10.3390/ijms21228523 (PMC7697731; doi:10.3390/ijms21228523)

**Supplementary Information**

**Figure S1.** Spectral absorbance of D-DIBOA quelated with decreasing concentrations of FeCl_3_ in acidic-water (pH<1).


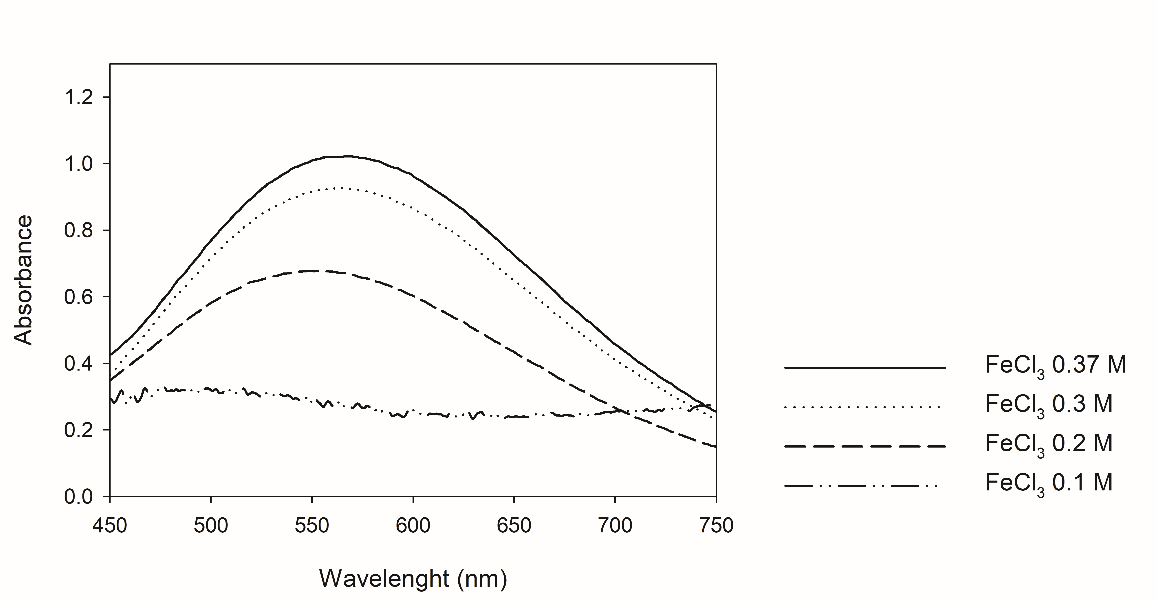

Supplement: Supplementary file 1 [file ijms-21-08523-s001.zip › ijms-978721-supplementary.docx]
